# Mapping the global research landscape and trends of older people living alone: a bibliometric analysis

**DOI:** 10.3389/fragi.2025.1524673

**Published:** 2025-07-03

**Authors:** Yu-Dan Wu, Jia-Xin Dong, Fu-Min Yu, Zhe-Hao Dong, Wei Ma, Yue Cai, Yu-Qing Cai, Yang Mu, Xiang Cui, Yi-Ran Wang, Hui-Jun Li, Xiao-Tao Yang, Duo-Ning Yuan, Shuang Wang, Nuo Cheng, Guang-Wei Zhang

**Affiliations:** ^1^ Department of General Practice, The First Hospital of China Medical University, Shenyang, China; ^2^ School of Nursing, China Medical University, Shenyang, China; ^3^ Department of Cardiovascular Surgery, Guangdong Provincial People’s Hospital, Guangdong Academy of Medical Sciences, Southern Medical University, Guangzhou, China; ^4^ School of Stomatology, China Medical University, Shenyang, China; ^5^ Department of Cardiology, The First Hospital of China Medical University, Shenyang, Liaoning, China; ^6^ Director of Research Services, Goodwill Information Technology Co., Ltd., Beijing, China; ^7^ Shenyang Medical & Film Science and Technology Co. Ltd., Shenyang, China; ^8^ Department of Smart Hospital Management, The First Hospital of China Medical University, Shenyang, China; ^9^ Enduring Medicine Smart Innovation Research Institute, Shenyang, China; ^10^ National Clinical Research Center for Laboratory Medicine, The First Hospital of China Medical University, Shenyang, China; ^11^ China Business School, Economics Faculty, Liaoning University, Shenyang, China; ^12^ Department of Gynecology, The First Hospital of China Medical University, Shenyang, China; ^13^ The Internet Hospital Branch of the Chinese Research Hospital Association, Beijing, China

**Keywords:** solitary, older adults, CiteSpace, social isolation, bibliometric analysis

## Abstract

**Background:**

The global aging trend is becoming increasingly severe, leading to a rise in the number of older adults living alone. As research on this population grows, a comprehensive analysis is essential.

**Objective:**

This study examines the current state of research on older adults living alone, identifies key trends and emerging topics, and provides a foundation for future investigations.

**Methodology:**

We conducted a subject search in the Web of Science (WOS) Core Collection database, retrieving articles related to older adults living alone based on titles, abstracts, and keywords from 1965 to 2024. Using CiteSpace (version 6.4.R1 Advanced), we generated collaborative networks among countries and authors, revealing research hotspots and frontiers in this field.

**Results:**

The study identified 740 relevant articles, showing an overall upward trend in publications. South Korea and China emerged as major contributors, though research remains decentralized. A total of 1,136 cited authors contributed to this field. Recent advances include the application of spatial recognition technology and artificial intelligence to prevent hazardous events among older adults living alone, highlighting a shift toward personalized and intelligent care solutions.

**Conclusion:**

This study demonstrates that older adults living alone represent an emerging focus in nursing research, yet international collaboration remains limited. The integration of intelligent devices and technologies to address caregiving challenges has become a prominent research hotspot in recent years.

## 1 Introduction

The global phenomenon of population ageing presents significant challenges. According to the World Health Organization (WHO), by 2030, one in six people worldwide will be aged 60 years or older. During this period, the proportion of the population aged 60 and above is projected to rise from 1 billion in 2020 to 1.4 billion. By 2050, the global population of individuals aged 60 and older is expected to double, reaching 2.1 billion. Among those aged 65 and above, an increasing proportion live alone, defined as those without spouses or children for companionship. This trend is becoming more prevalent across various countries due to demographic shifts and evolving social structures.

In Australia, approximately 25.4% of individuals aged 65 and over live alone (Rodwell, 2022). Similarly, in South Korea, the number of older adults living alone surged to 1.379 million in 2015, reflecting a 1.8-fold increase since 2005, with projections estimating a rise to 3.43 million by 2035—constituting 23.2% of the older adult population (Byeon, 2021a). In Japan, more than 15% of older adults lived alone as of 2005 (Sun et al., 2007). Both cross-sectional and longitudinal studies (Blanchard et al., 2003) indicate that older adults living alone face a higher risk of disability, mental health issues, and cognitive decline compared to those living with spouses or others (Koukouli et al., 2002; van Gelder et al., 2006; You and Lee, 2006). Due to their heightened need for social support and long-term care, this specific population encounters challenges such as difficulty managing emergencies, loneliness, and daily living struggles. Consequently, developing tailored health management strategies for older adults living alone—based on their unique characteristics and needs—has become a pressing societal concern (Baines and Hollingsworth, 1955).

In recent years, the growing population of older adults living alone has attracted increasing research attention, gradually establishing this as an emerging research focus. However, a systematic and comprehensive analysis of this field remains lacking. Bibliometrics, a branch of informatics, examines document structures and quantitative characteristics to perform both quantitative and qualitative analyses (Ninkov et al., 2022). This approach enables measurement of profile distributions, relationships, and clustering within research fields while comparing contributions across authors, institutions, countries, and journals (Hassan and Duarte, 2024). Recognized as crucial for developing guidelines, identifying research hotspots, and assessing trends, bibliometrics provides valuable insights into resource management, research collaboration, developmental trajectories, and journal impacts - making it particularly suitable for analyzing research concerning older adults. For example, Hong et al. (2022) conducted a bibliometric analysis of smart home technologies for older adults, demonstrating how well-designed smart homes can support aging-in-place preferences while generating economic benefits through reduced care expenditures. Similarly, Marziali et al. (2024) performed a bibliometric review of voice assistant technologies, highlighting their potential to mitigate loneliness and social isolation among older adults. Additional applications include, Zhang et al. (2023)’s investigation of oral health’s relationship to quality of life in aging populations, and Tan et al. (2021)’s examination of cardiovascular disease’s association with sarcopenia and subsequent disability risks. Given the distinct challenges faced by older adults living alone - including reduced quality of life, increased mental health vulnerability (Park et al., 2023a; Park et al., 2023b), and limited social support networks (Yeh and Lo, 2004), - a comprehensive bibliometric analysis of this field is urgently needed. Such an analysis would provide valuable insights into current research trends and facilitate better understanding of developments concerning this vulnerable population.

The advancement of electronic technologies, including infrared wireless sensors and artificial intelligence, has led to increasing applications of smart devices such as wearable bracelets and smart speakers (Park and Kim, 2022) for monitoring older adults living alone and preventing falls and other accidents (O’Brien et al., 2020; Alharbi et al., 2023). This study employs CiteSpace (version 6.4.R1 Advanced) to examine global research trends, core research foci, and scientific frontiers concerning older adults living alone. Through analysis of recent literature and citation networks, we identify leading research institutions, their collaborative networks, and emerging thematic trends in this field. Using bibliometric methods, we systematically analyze current research progress on older adults living alone. Our study aims to establish a solid scientific foundation for future research, with the dual objectives of expanding scholarly understanding and facilitating further investigations in this important area.

## 2 Methods

### 2.1 Data Sources and literature selection

We conducted our literature search using the Web of Science (WOS) Core Collection database. To ensure comprehensive coverage, we implemented a Topic Search (TS) strategy encompassing titles, abstracts, and keywords. The selection criteria for included literature were: (1) publication date prior to 31 December 2024; (2) focus on older adults living alone; (3) English-language publications; and (4) peer-reviewed articles and review papers. The literature search was finalized on 31 December 2024. Complete search strategies are presented in Supplementary Table S1.

### 2.2 Visual analysis software

The CiteSpace (version 6.4.R1 Advanced), a Java-based application developed by Dr. Chaomei Chen at Drexel University, is specifically designed for co-occurrence and co-citation analysis to visualize research trends and scientific frontiers. This software facilitates data importation, node selection, parameter configuration, and network visualization, enabling researchers to identify key research hotspots, emerging trends, and pivotal nodes within a given field.

### 2.3 Data analysis

We exported complete bibliographic data, including full article details and citations, from the Web of Science (WOS) Core Collection. For data processing efficiency, we exported records in plain text (TXT) format and imported them into CiteSpace for analysis. This dataset enabled the generation of scientific knowledge maps that visualize knowledge processing, structural relationships, and thematic evolution within the research domain. Our analysis employed a time-slicing approach from 1 January 1965 to 4 March 2024, with annual segmentation to examine longitudinal trends.

To identify evolving research themes, we analyzed term bursts within the literature. Node centrality indices were calculated to evaluate the relative importance of research concepts within the network, based on both the quantity and quality of connections. Higher centrality values indicate more influential nodes that play pivotal roles in information dissemination and research direction. These calculations utilized co-citation relationships in CiteSpace (version 6.2.R6 Advanced).

We implemented several analytical refinements: (1) a Top N = 50 threshold to focus on the most significant items per time slice; (2) the Pathfinder network pruning algorithm to enhance network clarity; and (3) Kleinberg’s (Kleinberg, 2002) to pinpoint emerging research frontiers, thereby improving our understanding of current developments and future trajectories in the field.

## 3 Results

### 3.1 Distribution of articles by publication years

**FIGURE 1:**
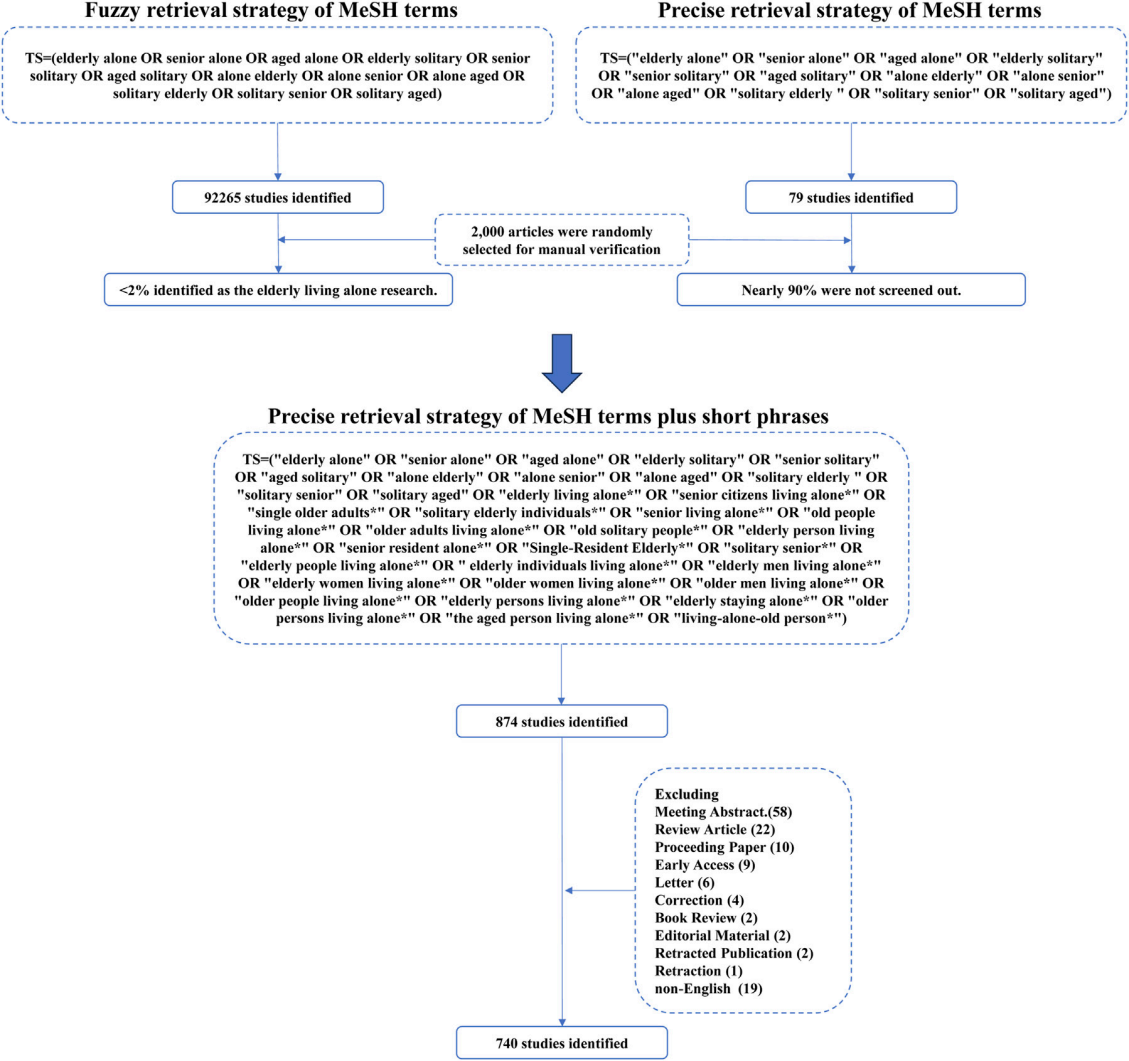
The flow diagram.

**FIGURE 2:**
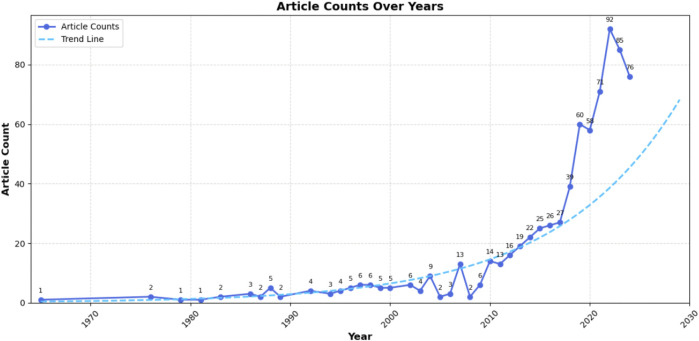
The global trend of study of older people living alone publication from 1965 to 2023.

**FIGURE 3:**
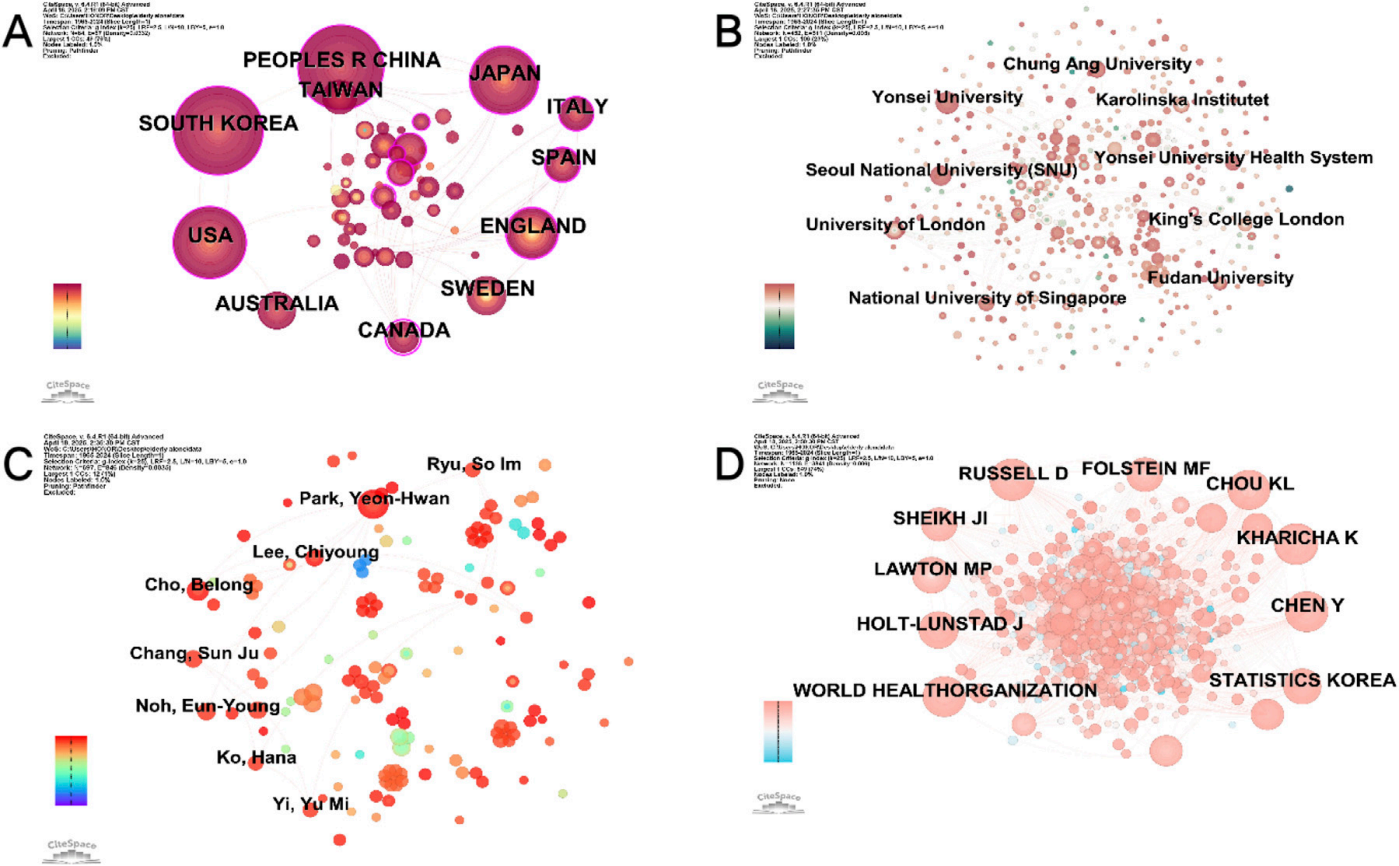
Distribution of countries/regions, authors and cited authors, institutions. **(A)** Map of country/regional cooperation network analysis. **(B)** Map of institution cooperation network analysis. **(C)** Map of author cooperation network analysis. **(D)** Map of cited author analysis.

Based on relevant MeSH terms related to older adults living alone, we initially conducted a broad search using a fuzzy retrieval strategy, which yielded 92,264 research articles. Given the excessive number of results, we randomly selected 2,000 articles for manual screening and found that only 38 met the inclusion criteria for studies specifically focused on older adults living alone. Subsequently, we applied a more targeted search strategy, which identified only 78 relevant articles. To ensure a more comprehensive and accurate inclusion of literature in this field, we further analyzed thematic descriptions related to older adults living alone and employed a refined retrieval strategy combining precise terms with short phrases. This approach ultimately resulted in the inclusion of 740 eligible articles. The detailed screening process is illustrated in Figure 1. The annual publication trend (Figure 2) exhibits a fluctuating yet overall upward trajectory, indicating that “older adults living alone” has progressively gained recognition as a significant societal issue, attracting growing scholarly attention. Research on this population has evolved into a distinctive feature of contemporary aging studies. The international collaboration network (Figure 3A) revealed a high-density cooperative pattern (n = 64, E = 67, density = 0.0332). Here, the E-value serves as an indicator of research novelty, quantifying breakthrough potential, while density reflects keyword co-occurrence strength, representing thematic cohesion within the field. These findings underscore robust international collaboration in this domain, emphasizing the collective nature of global research efforts and transnational cooperation trends in studies on older adults living alone.

### 3.2 Country/Region analysis

Among the 740 publications focusing on older adults living alone, researchers from at least 64 countries contributed to the literature. Table 1 lists the top 10 countries by publication volume. South Korea leads with 144 publications, though its centrality index (0.02) suggests limited international collaboration. China ranks second with 139 publications and a centrality index of 0.1, reflecting its strong position in the research network. The United States (122 publications, centrality 0.24), Japan (82 publications, centrality 0.12), and the United Kingdom (55 publications, centrality 0.19) also play prominent roles, demonstrating active engagement in this field. Australia, Taiwan, Canada, Sweden, Spain, and Italy occupy the sixth to 10th positions, with varying publication counts and centrality indices. Notably, Australia’s low centrality (0.01) indicates minimal influence, whereas Canada’s high centrality (0.23) underscores its pivotal role in international collaborations on this topic.

**TABLE 1:** Top 10 countries/regions contributing to publications.

Rank	Country/Regions	Centrality	Frequency
1	South Korea	0.02	144
2	Peoples R. China	0.1	139
3	USA	0.24	122
4	Japan	0.12	82
5	England	0.19	55
6	Australia	0.01	33
7	China Taiwan	0.09	30
8	Canada	0.23	28
9	Sweden	0.18	25
10	Spain	0.1	21
10	Italy	0.04	21

### 3.3 Institutional analysis

A total of 452 institutions worldwide have conducted research on older individuals living alone, but only four have published more than 10 papers (Supplementary Table S2). Among these, Yonsei University leads with 21 publications, followed by Seoul National University (SNU) with 19. The institutional research network exhibits low density (n = 452, E = 511, density = 0.005) (Figure 3B), suggesting limited collaboration and highlighting the need for stronger inter-institutional cooperation.

### 3.4 Distribution of authors

A total of 697 authors have contributed to research on older adults living alone, with the top 11 most prolific authors listed in Supplementary Table S3. Park YH ranks first with 12 articles, demonstrating significant academic leadership in this field. Cho B follows closely with six articles, securing second place and reflecting substantial research contributions. These findings highlight the prominent role of these authors while emphasizing the international and diverse nature of research in this field.

The author collaboration network (Figure 3C) exhibits low density (n = 697, E = 846, density = 0.0035), suggesting limited connectivity despite some cooperative efforts. This implies that while prolific authors collaborate to some extent, broader research networks remain underdeveloped and require further strengthening.

### 3.5 Co-citation network analysis

Co-citation network analysis (Figure 3D) revealed a low-density collaboration pattern (n = 1,136, E = 3,841, density = 0.006), indicating limited connectivity among cited authors. These findings suggest that while certain core researchers have made significant contributions, the overall collaborative network remains fragmented, highlighting opportunities for enhanced interdisciplinary cooperation to advance the field.

### 3.6 Keyword Co-occurrence analysis

Keyword co-occurrence analysis provides a methodological approach for identifying research hotspots within academic fields. Consistent with annual publication trends, this study analyzed keywords extracted from titles and abstracts of 740 articles, producing a co-occurrence network with 582 nodes and 2,896 links (Figure 5A). The relatively sparse co-citation links compared to other bibliometric analyses (Tan et al., 2023) suggest weaker inter-article connections, lower thematic coherence, and more dispersed research patterns in studies of older adults living alone. This pattern further indicates limited collaboration among researchers in this field.

Table 2 lists the top 10 most frequent keywords. “Health” appears as the predominant term, highlighting its central role in the discourse. “People” ranks second, while “older adults” places third but with lower centrality, reflecting its broad application across diverse research contexts. Other significant keywords include “living alone,” “loneliness,” and “adults,” demonstrating their frequent use in this research domain. Notably, “risk” shows strong associations with older adults living alone, typically denoting vulnerability and appearing across multiple research areas. CiteSpace’s keyword time zone visualization (Figure 5B) further illustrates the temporal evolution of these high-frequency keywords.

**TABLE 2:** Top 10 keywords Co-occurrence with the most frequency.

Rank	Keyword	Centrality	Frequency
1	Health	0.15	161
2	People	0.11	124
3	Older adults	0.06	114
4	Living alone	0.07	92
5	Loneliness	0.03	88
6	Adults	0.11	72
7	Risk	0.1	69
8	Social support	0.03	59
9	Depression	0.07	58
10	Mental health	0.06	56

### 3.7 Keyword burst analysis

**FIGURE 4:**
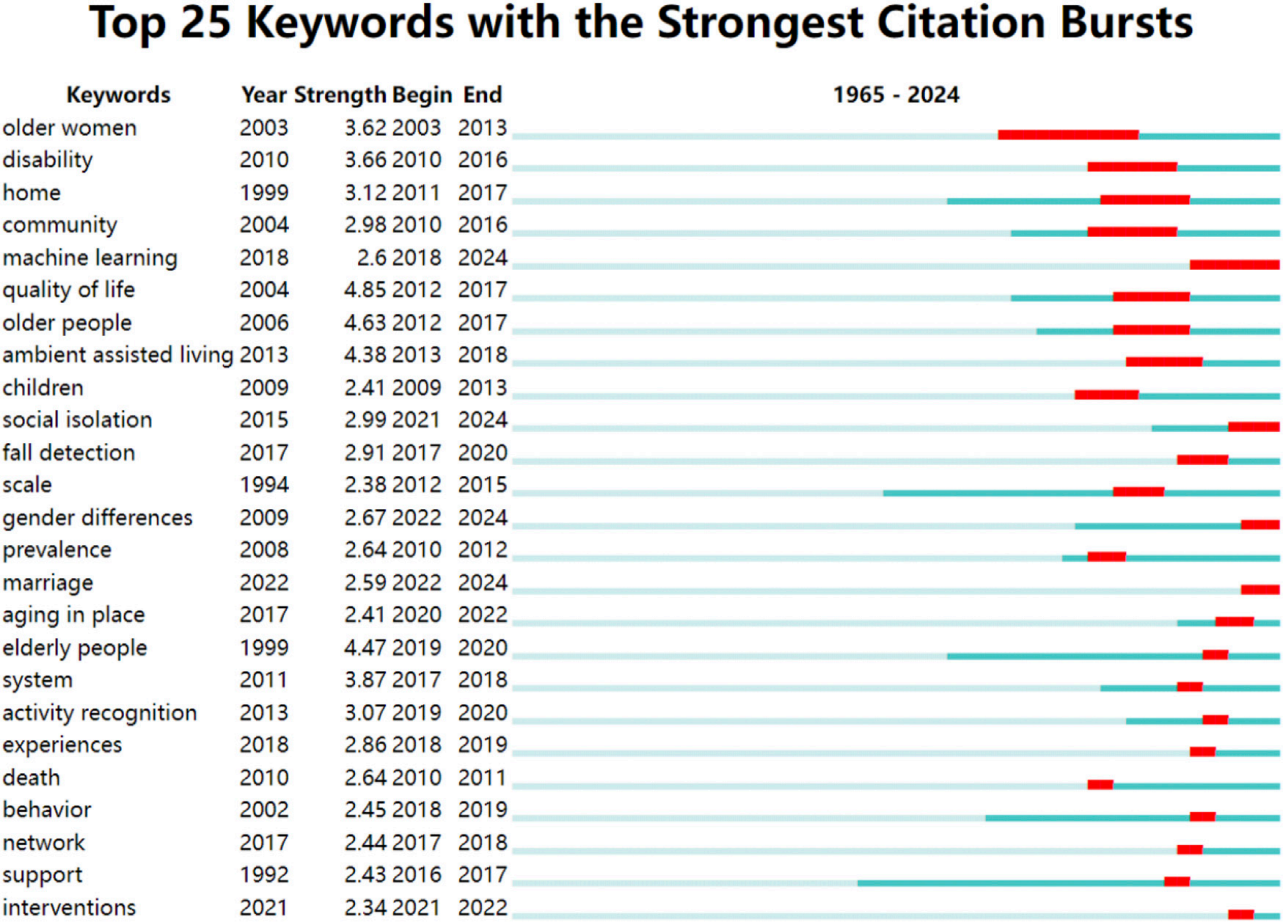
Top 25 keywords with the strongest citation bursts.

To identify emerging research trends in studies of older adults living alone, we analyzed keywords demonstrating significant citation bursts (Figure 4). The top twenty-five keywords with the highest burst intensity between 2003 and 2024 were identified, representing sudden increases in citation frequency that indicate shifting research priorities and scholarly developments.

“Older women” showed the earliest citation burst (2003–2013; intensity = 3.62), suggesting substantial research activity on this demographic during that period. Subsequently, “disability” emerged as a focus (2010–2016; intensity = 3.66), reflecting growing attention to disabled older adults. The concept of “quality of life” gained prominence (2012–2017), coinciding with increased scholarly emphasis on wellbeing in this population. More recently, “system” and “fall detection” exhibited citation bursts (2017–2018 and 2017–2020, respectively), highlighting the integration of smart technologies in aging research.

Most notably, “intervention” and “social isolation” experienced recent citation surges (2021–2022 and 2021–2024, respectively), underscoring heightened academic interest in psychological support and social connectivity for older adults living alone. These patterns collectively indicate a rapidly evolving field with expanding research directions.

### 3.8 Keyword Co-Citation cluster analysis

**FIGURE 5:**
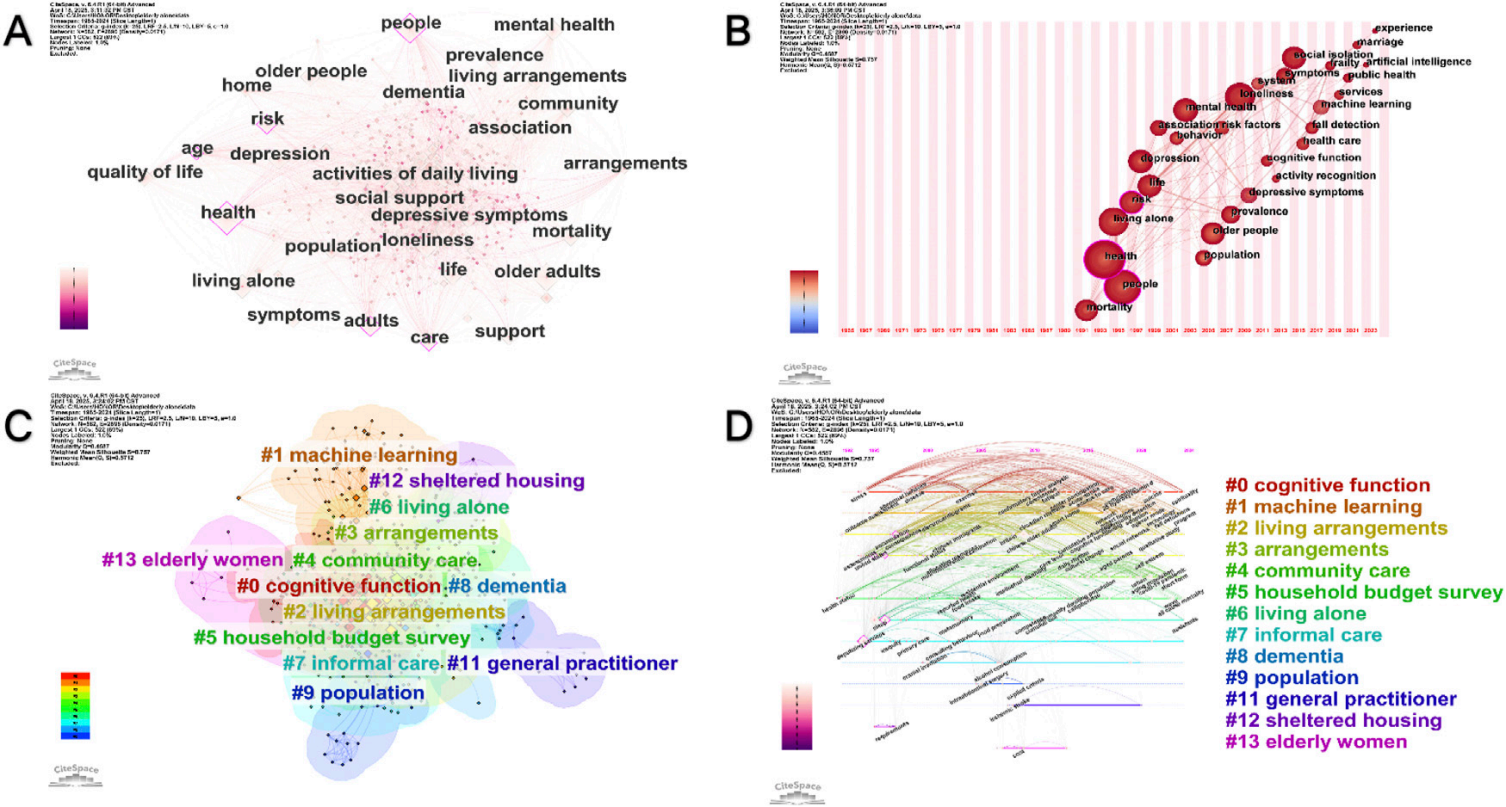
Keyword co-occurrence network analysis. **(A)** Map of keyword co-occurrence network analysis. **(B)** Keywords Time zone chart. **(C)** Keywords Cluster analysis map. **(D)** Keywords Time line view.

The log-likelihood ratio algorithm categorized 582 keywords into 14 distinct clusters (Figure 5C), with a mean silhouette value (S) of 0.757 for clusters 0–13. The S-index, which integrates citation and collaboration networks, provides a comprehensive measure of research impact. Each cluster was labeled using representative keywords, with “cognitive function” emerging as the largest cluster, indicating its prominence in research on older adults living alone. The second and third largest clusters were “machine learning” and “living arrangements,” respectively. Other clusters included “community care,” “dementia,” “population,” “general practitioner,” and “elderly women,” reflecting the diverse research trajectories within this field. These clusters are visualized in a timeline view for enhanced interpretation (Figure 5D).

**FIGURE 6:**
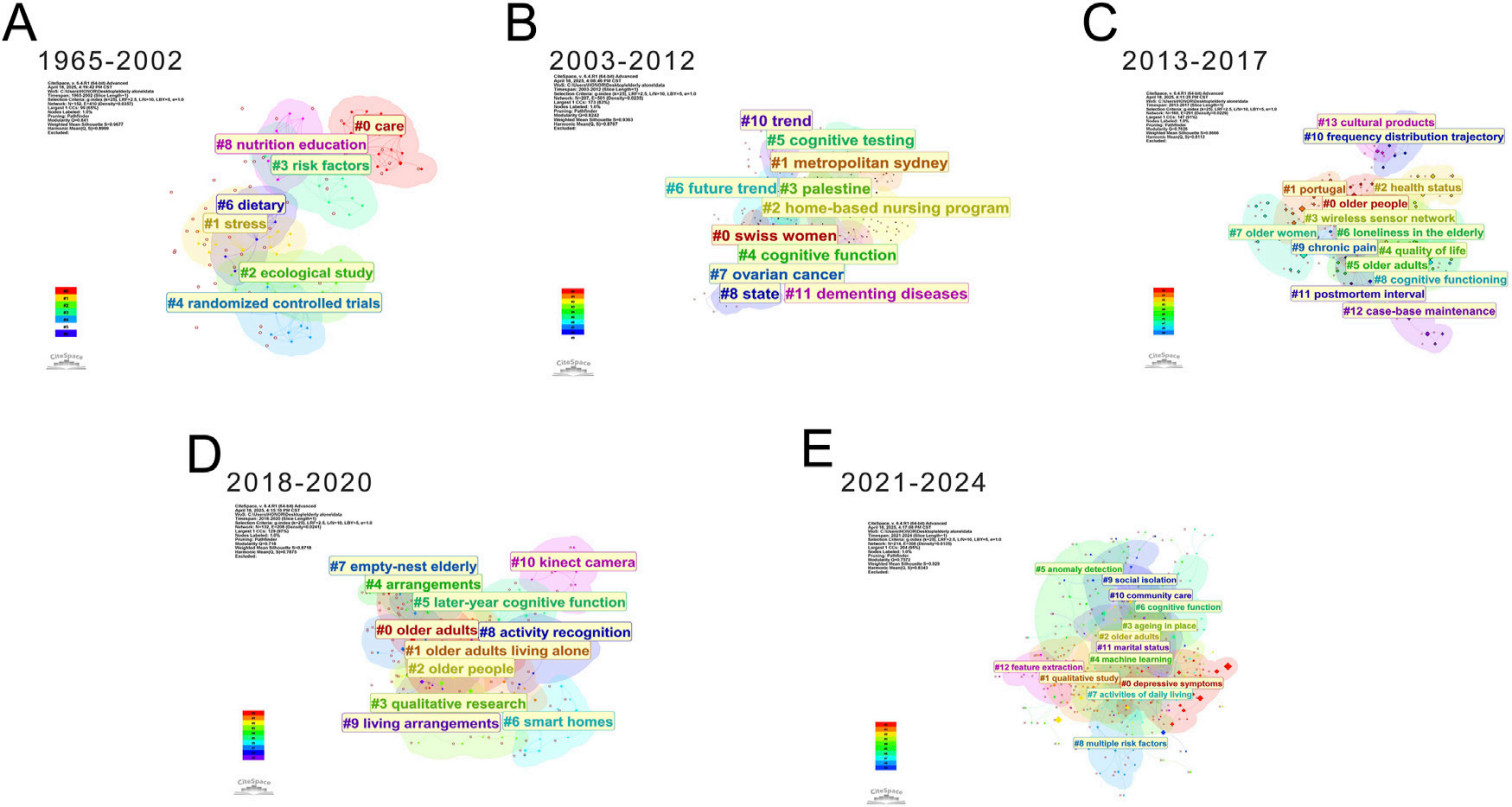
The keyword cluster analysis revealed five distinct chronological phases: **(A)** 1965–2002; **(B)** 2003–2012; **(C)** 2013–2017; **(D)** 2018–2020; and **(E)** 2021–2024.

A staged analysis of these clusters revealed distinct temporal patterns (Figure 6). From 1965 to 2002 (Figure 6A), research primarily examined basic aspects such as diet, nutrition education, and risk factors, predominantly through ecological studies and randomized controlled trials. Between 2003 and 2012 (Figure 6B), trend analysis gained prominence, focusing on cognitive testing and dementing diseases while expanding geographically to include regions like Sydney and Palestine. The period from 2013 to 2017 (Figure 6C) saw increased attention to quality-of-life factors, including health status, loneliness, and chronic pain, alongside the introduction of technologies like wireless sensor networks. From 2018 to 2020 (Figure 6D), research shifted toward living arrangements, late-life cognitive function, and smart home applications, incorporating activity recognition technologies. Most recently (2021–2024) (Figure 6E), studies have expanded into advanced domains such as community care and anomaly detection, employing multifaceted approaches to enhance elderly wellbeing. We analyzed the top five most frequent keywords across different categories (Table 3). In the emerging technologies category, the highest frequency keywords were: machine learning (n = 16), deep learning (n = 5), wireless sensor network (n = 3), artificial intelligence (n = 3), and ambient intelligence (n = 3). For disease conditions, the most common keywords were health (n = 161), depression (n = 58), mental health (n = 56), dementia (n = 23), and Alzheimer’s disease (n = 10). Regarding research methods, the top keywords included prevalence (n = 40), association (n = 36), risk factors (n = 24), validity (n = 18), and thematic analysis (n = 2). In the intervention variables category, living alone appeared most frequently (n = 92), followed by social support (n = 59), care (n = 52), quality of life (n = 46), and physical activity (n = 30). For intelligent systems, the top terms were activity recognition (n = 18), ambient assisted living (n = 12), smart homes (n = 4), fall detection (n = 2), and assistive robotics (n = 1).

**TABLE 3:** Top 5 keywords in each category.

Category	Keywords	Frequency
Emerging technologies	Machine learning	16
	Deep learning	5
	Wireless sensor network	3
	Artificial intelligence	3
	Ambient intelligence	3
Disease status	Health	161
	Depression	58
	Mental health	56
	Dementia	23
	Alzheimer’s disease	10
Research methods	Prevalence	40
	Association	36
	Risk factors	24
	Validity	18
	Thematic analysis	2
Intervention variables	Living alone	92
	Social support	59
	Care	52
	Quality of life	46
	Physical activity	30
Smart systems	Activity recognition	18
	Ambient assisted living	12
	Smart homes	4
	Fall detection	2
	Assistive robotics	1

## 4 Discussion

Our study analyzed the literature on older adults living alone from 1965 to 2024. The increasing trend in publications reflects sustained scholarly interest and advancements in research on solitary aging. Notably, publications grew gradually from 2008 to 2017 before rising sharply after 2017, indicating heightened academic and practical engagement with this topic. This trend aligns with the earlier rapid growth in the proportion of older adults living alone, underscoring the importance of synthesizing research in this field.

Globally, South Korea and China lead in research on solitary older adults, excelling in scientific output, publication volume, and academic influence. In South Korea, institutions such as Seoul National University (SNU) and Yonsei University have laid a strong foundation for this research area. In China, the high publication output and contributions from scholars like Chen Y at Fudan University highlight the country’s significant role. Although the United Kingdom produces fewer publications, institutions like the University of London play a key role in international collaborations. Conversely, the United States and Japan have substantial publication volumes but exhibit limited cross-border collaboration, with no single institution dominating domestic output.

Researchers such as Park YH, Chang SJ, and Byeon H have made prolific contributions, demonstrating substantial influence in the field. Park YH, for instance, advocates for community-based integrated service (CBIS) models grounded in Aging-in-Place (AIP) principles to better support the health and daily living needs of solitary older adults. Additionally, the highly cited works of Lawton MP and Kharicha K exemplify the impact of influential, high-quality research in advancing this discipline.

The keyword cluster analysis yielded several significant insights. Based on these findings, we further categorized the keywords and examined their relationships across five domains: emerging technologies, disease status, research methods, intervention variables, and smart systems. This comprehensive analysis provides a holistic perspective on current research trends while offering targeted directions for future studies. In the emerging technologies category, high-frequency terms such as “machine learning,” “ambient intelligence,” “deep learning,” and “wireless sensor network” indicate the growing prominence of AI-IoT integration in health monitoring and care for older adults living alone (Rajasekaran et al., 2009; Byeon, 2021b). The disease conditions category featured prevalent terms including “depression,” “mental health,” “dementia,” and “Alzheimer’s disease,” underscoring substantial academic focus on mental health and cognitive impairment among solitary older adults - a critical concern in aging societies (Cermakova et al., 2017; Tamminen et al., 2019; Koo et al., 2021). Analysis of research paradigms revealed common keywords like “prevalence,” “association,” “risk factors,” “validity,” and “thematic analysis,” demonstrating an evolution from descriptive studies to more sophisticated correlational and empirical investigations, marking methodological maturation in the field (Yang et al., 2023; Park et al., 2025). Among intervention variables, predominant terms such as “living alone,” “social support,” “care,” and “quality of life” highlight the centrality of social support systems in enhancing wellbeing for this population (Renwick et al., 2020). The intelligent systems category featured keywords including “activity recognition,” “ambient assisted living,” “smart homes,” “fall detection,” and “assistive robotics,” illustrating the transformative potential of technology-enabled home care solutions in addressing accessibility and sustainability challenges in elder care (Daniele et al., 2019).

Keywords that experience sudden increases in citations are recognized as indicators of emerging research themes or trends. Our analysis identified multiple research streams within the field of solitary older adults, encompassing a wide array of topics. The majority of studies concentrate on enhancing quality of life, family and social support, intelligent assistive technologies, and social isolation among older adults living alone. As emerging technologies continue to advance, smart devices are expected to play an increasingly prominent role in mitigating loneliness, improving quality of life, and preventing or detecting hazardous events such as falls.

Our keyword citation trend analysis reveals that global research hotspots on solitary older adults can be categorized into five distinct phases. Phase 1 (1965–2003) is characterized by a limited number of publications and weak thematic connections, which explains its absence from the burst keyword timeline.

Phase 2 (2003–2012). Research began focusing on gender-specific studies of solitary older adults, particularly older women, as reflected in keywords such as “older women” and “home.” Health disparities by gender were evident; for instance, a cross-sectional study in Taiwan found that women aged 65 and older living alone faced a 1.6 times higher risk of depression than their male counterparts, especially those over 85 with limited social support (Lin and Wang, 2011). Gender differences also emerged in coping with memory loss, with women with Alzheimer’s disease actively reconstructing self-awareness (Frazer et al., 2012). Familial dependence was pronounced, with many older adults preferring to age and die at home, influenced by their physical environment, socioeconomic conditions, and access to care (Rolls et al., 2011). This phase thus emphasized more nuanced, gender-sensitive research.

Phase 3 (2013–2017). Research shifted toward enhancing quality of life and societal support for solitary older adults, as indicated by keywords like “support,” “quality of life,” “disability,” and “ambient assisted living.” A central focus was maintaining independence despite health challenges. Studies highlighted that depression and social isolation were more prevalent among those lacking support, leading to poorer quality of life (Lee and Kim, 2014; Parlar Kilic et al., 2014; Arslantas et al., 2015; McHugh et al., 2015). Effective interventions mitigated loneliness and improved dietary, physical, and mental health (Bilotta et al., 2012; McHugh et al., 2015). Disability research advocated for better healthcare access and non-emergency transport to prevent treatment delays (Henning-Smith et al., 2016).

Phase 4 (2018–2020). This phase was marked by the rise of electronic and wearable technologies, with keywords such as “fall detection,” “network,” “system,” and “machine learning.” Smart devices leveraging IoT, sensors, and AI enhanced safety and quality of life (Huh, 2018; Wang et al., 2019).

Phase 5 (2021–2024). Attention returned to physical and mental health, with keywords like “social isolation,” “aging in place,” and “intervention.” AI advancements enabled precise applications for improving social support and quality of life, addressing heightened risks of cognitive decline and depression (Lee et al., 2020). Technologies such as infrared sensors (Wang et al., 2022; Yang et al., 2022), IoT (Luperto et al., 2023), deep learning (Khraief et al., 2020), and wearable devices has progressed rapidly, providing tools for social support and cognitive improvement (Choi et al., 2022). AI-based home devices alleviated depression and loneliness (Park and Kim, 2022; Yan et al., 2024). while psychological and neurodegenerative issues received renewed focus (Parlar Kilic et al., 2014; Breaz, 2019; Charpentier and Kirouac, 2022). Strategies to improve life satisfaction were urged (Hou and Zhang, 2023). Non-technological factors, like pet ownership, were also explored; a cohort study found pets slowed verbal memory decline (Li et al., 2023). Another study further indicated that pet companionship may enhance wellbeing in this population, thereby mitigating feelings of loneliness and cognitive decline (Stanley et al., 2014). Notably, research employing AI-powered robotic pets has shown that integrating these companions into daily life—while iteratively refining their design based on user feedback—can effectively reduce loneliness and facilitate social engagement among isolated older adults with limited social connections (Hudson et al., 2020). Sum up this phase emphasized intelligent technologies and cognitive health in elder care.

Several crucial research hotspots—including “living arrangements,” “living alone,” “daily rhythm,” “intervention,” “climate change,” “care,” “general practitioner,” “elderly caregiving microsystem,” and “nutrient intakes”—are not prominently featured in visual maps but remain significant. Among these, studies on malnutrition, living arrangements, and daily rhythms are particularly noteworthy. Malnutrition represents a global health risk for older adults. A Norwegian cross-sectional study revealed that older adults living alone struggle to maintain adequate nutrition through self-care activities (Tomstad et al., 2012), highlighting the need for healthcare professionals to identify at-risk individuals and provide nutritional guidance. Furthermore, loneliness is a significant predictor of anorexia nervosa, malnutrition, and malnutrition risk (Ramic et al., 2011), suggesting that reducing loneliness may improve both nutritional intake and quality of life.

Consistent evidence indicates that older adults living alone have poorer nutritional intake than their married counterparts (Pequignot et al., 1967; Yamagami et al., 1998; Charlton, 1999; Hsieh et al., 2010; Tomstad et al., 2012). For instance, an Israeli policy survey found that low-income solitary men consumed fewer vegetables, fruits, and vitamin C than those with partners (Guggenheim and Margulec, 1965). Given that vitamin C deficiency increases scurvy risk, living alone emerges as an independent risk factor for poor nutrition in older adults (Oulie, 1988). Given that vitamin C deficiency increases scurvy risk, living alone emerges as an independent risk factor for poor nutrition in older adults.

Positive living arrangements and structured daily rhythms significantly influence the physical and mental health of older adults living alone. Daily activities typically encompass social interactions, physical exercise, hobbies, and community-based engagements (Cederbom et al., 2014). Research indicates that solitary older adults tend to exhibit lower physical activity levels and higher body fat percentages, increasing their susceptibility to hypertension, deep vein thrombosis, and other cardiovascular conditions (Kong and So, 2017). Implementing regular physical activity regimens, such as Tai Chi, Baduanjin, and walking, has been shown to enhance mental health, reduce loneliness and depression, and strengthen social connections, thereby fostering a greater sense of dignity among this population (Wu et al., 2024). Intervention programs like forest therapy in Korea have demonstrated dual benefits by addressing psychological loneliness while promoting healthier lifestyles, consequently reducing dementia risk (Jin and Son, 2018b; Jin and Son, 2018a). Similarly, structured community activities—including group exercises and art projects—have proven effective in improving both physical and mental wellbeing (Kil et al., 2018; Kim et al., 2018; Aydin and Kutlu, 2021; Beselt et al., 2023). Collectively, these findings underscore the critical role of maintaining positive living arrangements and consistent daily routines, which not only enhance functional independence but also facilitate meaningful social interactions. Such insights highlight daily schedules and living environments as viable targets for interventions aimed at supporting solitary older adults.

In summary, our bibliometric analysis of literature spanning 1965 to 2024 elucidates key trends and emerging themes in research on older adults living alone. This synthesis underscores the field’s evolution, advocating for multidisciplinary collaboration and innovative integration of intelligent technologies to address this population’s complex needs. By combining supportive living environments with proactive healthcare strategies, future efforts can deliver more compassionate and effective care solutions.

## Data Availability

The original contributions presented in the study are included in the article/Supplementary Material, further inquiries can be directed to the corresponding authors.
