# Supplementary material for: Mapping the global research landscape and trends of older people living alone: a bibliometric analysis
Source: Front Aging. 2025 Jul 3;6:1524673. doi: 10.3389/fragi.2025.1524673 (PMC12268210; doi:10.3389/fragi.2025.1524673)
Supplement: Supplementary file 1 [file Table1.docx]

Supplementary tables

Supplementary Table 1: Search strategies for literature

| Search number | Search Strategies | Literature number |
| --- | --- | --- |
| #1 | TS=(elderly alone OR senior alone OR aged alone OR elderly solitary OR senior solitary OR aged solitary OR alone elderly OR alone senior OR alone aged OR solitary elderly OR solitary senior OR solitary aged) | 92265 |
| #2 | TS=("elderly alone" OR "senior alone" OR "aged alone" OR "elderly solitary" OR "senior solitary" OR "aged solitary" OR "alone elderly" OR "alone senior" OR "alone aged" OR "solitary elderly " OR "solitary senior" OR "solitary aged") | 79 |
| #3 | TS=("elderly alone" OR "senior alone" OR "aged alone" OR "elderly solitary" OR "senior solitary" OR "aged solitary" OR "alone elderly" OR "alone senior" OR "alone aged" OR "solitary elderly " OR "solitary senior" OR "solitary aged" OR "elderly living alone*" OR "senior citizens living alone*" OR "single older adults*" OR "solitary elderly individuals*" OR "senior living alone*" OR "old people living alone*" OR "older adults living alone*" OR "old solitary people*" OR "elderly person living alone*" OR "senior resident alone*" OR "Single-Resident Elderly*" OR "solitary senior*" OR "elderly people living alone*" OR " elderly individuals living alone*" OR "elderly men living alone*" OR "elderly women living alone*" OR "older women living alone*" OR "older men living alone*" OR "older people living alone*" OR "elderly persons living alone*" OR "elderly staying alone*" OR "older persons living alone*" OR "the aged person living alone*" OR "living-alone-old person*") | 740 |

Supplementary Table 2: Top 10 Institutions With the Most Publications

| Rank | Institution | Centrality | Frequency |
| --- | --- | --- | --- |
| 1 | Yonsei University | 0.01 | 21 |
| 2 | Seoul National University (SNU) | 0.01 | 19 |
| 3 | University of London | 0.06 | 18 |
| 4 | National University of Singapore | 0.06 | 12 |
| 5 | Chung Ang University | 0 | 11 |
| 6 | Karolinska Institutet | 0.01 | 10 |
| 6 | Monash University | 0 | 10 |
| 6 | Yonsei University Health System | 0 | 10 |
| 9 | Fudan University | 0 | 9 |
| 9 | King's College London | 0.01 | 9 |
| 10 | Catholic University of Korea | 0 | 8 |
| 10 | Chinese University of Hong Kong | 0.02 | 8 |
| 10 | Kyung Hee University | 0 | 8 |
| 10 | Tokyo Metropolitan Institute of Gerontology | 0 | 8 |
| 10 | University of California System | 0 | 8 |
| 10 | University of Hong Kong | 0.01 | 8 |

Supplementary Table 3: Top 10 Authors with The Most Publications

| Rank | Author | Country | Institution | Article Counts |
| --- | --- | --- | --- | --- |
| 1 | Park, Yeon-Hwan | SOUTH KOREA | Seoul National University (SNU) | 12 |
| 2 | Cho, Belong | SOUTH KOREA | Seoul National University (SNU) | 6 |
| 3 | Johansson, SE | SWEDEN | Lund University | 4 |
| 3 | Hicks, Allan | USA | Int Pacific Halibut Commiss | 4 |
| 3 | Lee, Chiyoung | USA | University of Arizona | 4 |
| 3 | Noh, Eun-Young | SOUTH KOREA | Konkuk University | 4 |
| 3 | Chen, Yu | PEOPLES R CHINA | Fudan University | 4 |
| 3 | While, Alison E | ENGLAND | King's College London | 4 |
| 3 | Chang, Sun Ju | SOUTH KOREA | Seoul National University (SNU) | 4 |
| 3 | Byeon, Haewon | SOUTH KOREA | Inje University | 4 |
| 3 | Sundquist, J | SWEDEN | Lund University | 4 |
